# Management and Reconstruction of a Gastroesophageal Junction Adenocarcinoma Patient Three Years after Pancreaticoduodenectomy: A Surgical Puzzle

**DOI:** 10.1155/2016/5650382

**Published:** 2016-08-18

**Authors:** Dionysios Dellaportas, James A. Gossage, Andrew R. Davies

**Affiliations:** Department of Oesophagogastric Surgery, St Thomas' Hospital, King's College London, London, UK

## Abstract

*Introduction*. With the improving survival of cancer patients, the development of a secondary primary cancer is an increasingly common phenomenon. Extensive surgery during initial treatment may pose significant challenges to surgeons managing the second primary cancer.* Case Presentation*. A 69-year-old male, who had a pancreaticoduodenectomy three years ago for pancreatic head adenocarcinoma, underwent an uneventful extended total gastrectomy for gastroesophageal junctional adenocarcinoma. The reconstruction controversies and considerations are highlighted.* Discussion*. Genetic, environmental, and lifestyle factors are common for several gastrointestinal malignancies. However, the occurrence of a second unfavorable cancer such as gastroesophageal adenocarcinoma after pancreatic head cancer treatment is extremely uncommon. This clinical scenario possesses numerous difficulties for the surgeon, since surgical resection is the mainstay of treatment for both malignancies. Gastrointestinal reconstruction becomes challenging and requires careful planning and meticulous surgical technique along with sound intraoperative judgement.

## 1. Introduction

Improvement of management for cancer patients, increased overall survival, and identification of the disease in early stages have led to the rare event of patients developing a second primary cancer later in their lives. In various studies the incidence of second primary cancer is 6.6–9% [1]. Complex surgery during initial cancer treatment can limit surgical options during management plan of the secondary malignancy. We present an interesting case of a 69-year-old male treated successfully in our institution, who developed gastroesophageal junction adenocarcinoma three years after pancreaticoduodenectomy for pancreatic head adenocarcinoma and finally underwent an extended total gastrectomy. The aim of our study is to highlight the treatment options, the controversies, and the challenging surgical reconstructive options in such a clinical scenario.

## 2. Case Presentation

A 69-year-old white male patient was investigated for dysphagia and vomiting associated with weight loss. The patient had a medical history of hypertension and hyperlipidemia and a surgical history of pylorus-preserving pancreaticoduodenectomy three years ago for pancreatic head adenocarcinoma. The latter's histopathological stage was pT1N0 (TNM 7th Edition AJCC classification) and the patient received no adjuvant treatment. Upper gastrointestinal endoscopy revealed an ulcerated, friable tumour of the gastroesophageal junction (GOJ) and histopathology confirmed the presence of poorly differentiated adenocarcinoma. Formal staging of the disease was performed with computed tomography (CT) scan, followed by positron emission tomography (PET/CT) scan and endoscopic ultrasound (EUS) and after multidisciplinary team (MDT) discussion the patient was staged as Siewert type II cT2N1M0 GOJ adenocarcinoma [2]. No evidence of distant disease from either malignancy was revealed and the patient was considered for radical treatment. According to usual management [3] he received three cycles of neoadjuvant chemotherapy with epirubicin, cisplatin, and capecitabine (ECX protocol), which was well tolerated and with good radiological and clinical response. Restaging CT scan demonstrated regression of the two enlarged left-gastric lymph nodes. The patient was offered either surgery or definite chemoradiotherapy for locoregional control of the disease and chose the former option after extensive counselling as to the associated risks and benefits.

Surgical planning raised some anatomical challenges. Reviewing the pancreaticoduodenectomy reconstruction method from his operative note three years ago, the patient had a pylorus-preserving pancreaticoduodenectomy (PPPD) with an isolated Roux-en-Y reconstruction for the pancreaticojejunal anastomosis. Ligation of the gastroduodenal artery (GDA) was done during PPPD, which gives rise to the right gastroepiploic artery precluding the use of the stomach as conduit for any kind of oesophagogastrectomy. Moreover, the use of colon as a conduit is appealing, but the use of either the left or the right colon involves dissection of the middle colic vessels and meticulous preservation of anastomotic arcades, in an area where the isolated jejunal Roux limb may ascend, retrocolic, and to the left of the above vessels for the pancreaticojejunal anastomosis. Extensive adhesiolysis may be anticipated since the superior mesenteric vessels, which give rise to the middle colic vessels, were dissected during the PPPD.

With all the above in mind, surgical exploration was decided and performed through the old bilateral subcostal incision. The primary tumour involved the GOJ, but after transhiatal mobilization and intraoperative upper gastrointestinal endoscopy it became feasible to get to macroscopically clear margins to the thoracic oesophagus without the need of thoracotomy. In addition, duodenojejunostomy was antecolic and performed on the long blind ended jejunal loop that originated just below the duodenojejunal flexure from the previous Whipple procedure. After division of the jejunum, an extended total gastrectomy was performed. Oesophagojejunal anastomosis was the only reconstructive anastomosis needed in this complex case to reestablish gastrointestinal continuity. Total operative time was 170 minutes. Final histopathology showed a complete resection (R0) of a ypT1bN0 GOJ poorly differentiated adenocarcinoma, with negative lymph nodes (0/29). The patient had an uneventful postoperative course and was discharged on the 10th postoperative day, while MDT decision was for no adjuvant treatment. He remains well on postoperative surveillance.

## 3. Discussion 

The development of a second malignancy as mentioned above is an unusual event, which is increasingly common in an era of improved survival for cancer patients. Particularly for patients with unfavorable malignancies, like pancreatic, oesophageal, or gastric cancer, the incidence of second primary cancer is less than 5% [1]. Simultaneous or metachronous occurrence of gastroesophageal adenocarcinoma with periampullary tumours is extremely unusual. In an interesting review of more than 10.000 gastric cancer patients [4], 96 of them had a second primary malignancy and only 5 of them had pancreatic adenocarcinoma. For oesophageal adenocarcinoma patients, the most common second primary malignancies are gastric, oropharyngeal, and lung cancer, while pancreatic cancer is extremely rare [5]. Obviously, common risk factors such as genetic, lifestyle, or dietary factors can give rise to gastrointestinal malignancies of different sites, and if the patient survives long enough after an initial treatment, a second primary gastrointestinal cancer may develop. Surgical resection remains the mainstay of treatment for pancreatic, gastric, and oesophageal cancer.

Technically, surgical strategies have been described in the setting of previous oesophagogastrectomy for the performance of pancreaticoduodenectomy [6], mostly involving the use of colon as conduit, and in the form of case reports. Other challenging options when the right gastroepiploic vessels are compromised include the use of supercharged jejunal interposition conduits [7] or microvascular reconstruction methods for the right gastroepiploic vessels [8, 9]. Oesophagogastrectomy and pancreaticoduodenectomy are individually extensive operations, and their combination either simultaneously or metachronously poses unique challenges. In a recent review of such cases, only three cases involved oesophageal resection after pancreaticoduodenectomy [10]. Our case involved an extended total gastrectomy and intrathoracic oesophagojejunal anastomosis, and to the best of our knowledge it is the first case reporting such a reconstruction configuration.

We suggest careful preoperative planning which includes understanding the method of reconstruction following the previous Whipple procedure and endoscopic assessment of the oesophagogastric primary by the surgical team themselves to establish its exact location. Having at one's disposal, a variety of surgical strategies for junctional tumours (e.g., extended total gastrectomy, left thoracoabdominal oesophagogastrectomy, substernal colonic interposition, and supercharged jejunal reconstruction methods) afford flexibility that may be crucial in determining success. Furthermore, it is probably unnecessary to dissect all of the jejununa to the pancreas and bile duct in these cases, which may lead to complications. The simplest option remains to use the loop of small bowel previously anastomosed to the stomach/duodenum and (staying close to the bowel wall during its division) mobilize this to reach the oesophagus. Failing this, the jejunum can simply be stapled off and either a Roux loop created distally (beyond the pancreatico- and hepaticojejunostomies) or a colon interposition is used. Postoperatively, nutritional monitoring plays a key role as these patients are at higher risk of the many side effects of complex gastrointestinal surgery such as pancreatic insufficiency, small intestinal bacterial overgrowth, and vitamin/mineral deficiencies.

The use of complex surgery in treatment of cancer patients has made reoperations challenging; however reconstruction options exist and meticulous preoperative planning, imaging tests, and intraoperative judgement help the surgeon to perform extensive and successful oncologically sound procedures.

## Abbreviations

GOJ: Gastroesophageal junction
CT: Computed tomography
PET/CT: Positron emission tomography/computed tomography
EUS: Endoscopic ultrasound
MDT: Multidisciplinary team
PPPD: Pylorus-preserving pancreaticoduodenectomy
GDA: Gastroduodenal artery
R0: Complete macroscopic/microscopic resection in clear margins.
